# Genetic loci for serum magnesium among African-Americans and gene-environment interaction at *MUC1* and *TRPM6* in European-Americans: the Atherosclerosis Risk in Communities (ARIC) study

**DOI:** 10.1186/s12863-015-0219-7

**Published:** 2015-05-29

**Authors:** Adrienne Tin, Anna Köttgen, Aaron R Folsom, Nisa M Maruthur, Salman M Tajuddin, Mike A Nalls, Michele K Evans, Alan B Zonderman, Christopher A Friedrich, Eric Boerwinkle, Josef Coresh, Wen Hong Linda Kao

**Affiliations:** Johns Hopkins Bloomberg School of Public Health, Baltimore, MD USA; University Medical Center Freiburg, Freiburg, Germany; University of Minnesota School of Public Health, Minneapolis, MN USA; Johns Hopkins University School of Medicine, Baltimore, MD USA; National Institute on Aging, National Institutes of Health, Bethesda, MD USA; University of Mississippi Medical Center, Jackson, MS USA; University of Texas School of Public Health, Houston, TX USA

**Keywords:** Gene-environment interaction, Serum magnesium, MUC1, TRPM6

## Abstract

**Background:**

Low serum magnesium levels have been associated with multiple chronic diseases. The regulation of serum magnesium homeostasis is not well understood. A previous genome-wide association study (GWAS) of European ancestry (EA) populations identified nine loci for serum magnesium. No such study has been conducted in African-Americans, nor has there been an evaluation of the interaction of magnesium-associated SNPs with environmental factors. The goals of this study were to identify genetic loci associated with serum magnesium in an African-American (AA) population using both genome-wide and candidate region interrogation approaches and to evaluate gene-environment interaction for the magnesium-associated variants in both EA and AA populations. We conducted a GWAS of serum magnesium in 2737 AA participants of the Atherosclerosis Risk in Communities (ARIC) Study and interrogated the regions of the nine published candidate loci in these results. Literature search identified the influence of progesterone on *MUC1* expression and insulin on *TRPM6* expression.

**Results:**

The GWAS approach in African-American participants identified a locus near *MUC1* as genome-wide significant (rs2974937, beta = −0.013, *p* = 6.1x10^−9^). The candidate region interrogation approach identified two of the nine loci previously discovered in EA populations as containing SNPs that were significantly associated in African-American participants (*SHROOM3* and *TRPM6*). The index variants at these three loci together explained 2.8 % of the variance in serum magnesium concentration in ARIC African-American participants. On the test of gene-environment interaction in ARIC EA participants, the index variant at *MUC1* had 2.5 times stronger association in postmenopausal women with progestin use (beta = −0.028, *p* = 7.3x10^−5^) than in those without any hormone use (beta = −0.011, *p* = 7.0x10^−8^, p for interaction 0.03). At *TRPM6,* the index variant had 1.6 times stronger association in those with lower fasting insulin levels (<80pmol/L: beta = −0.013, *p* = 1.6x10^−7^; ≥80pmol/L: beta = −0.008, *p* = 1.8x10^−2^, p for interaction 0.03).

**Conclusions:**

We identified three loci that explained 2.8 % of the variance in serum magnesium concentration in ARIC African-American participants. Following-up on functional studies of gene expression identified gene-environment interactions between progestin use and *MUC1* and between insulin and *TRPM6* on serum magnesium concentration in ARIC European-American participants. These results extend our understanding of the metabolism of serum magnesium.

**Electronic supplementary material:**

The online version of this article (doi:10.1186/s12863-015-0219-7) contains supplementary material, which is available to authorized users.

## Background

Low serum magnesium levels have been associated with multiple common chronic diseases, including cardiovascular diseases [1], hypertension [2], diabetes [3], and chronic kidney disease [4]. The regulation of serum magnesium hemostasis is not well understood. The heritability of serum magnesium is estimated to be 27 % [5], suggesting genetic association studies of serum magnesium can improve our understanding of serum magnesium metabolism. A genome-wide association study (GWAS) in populations of European ancestry (EA) previously identified nine loci associated with serum magnesium levels [6], and these loci explained <2 % of serum magnesium inter-individual variation [6]. African-Americans (AA) are known to have lower serum magnesium levels [7], and no results from GWAS in AA populations have been reported. It is known that associated variants may be ancestry-specific [8]. Gene-environment interactions can also modify genetic effects. The investigation of genetic associations across ethnic groups and the identification of gene-environment interactions have the potential to increase our understanding of the regulation of serum magnesium levels. The goals of the present study were to 1) identify genomic loci associated with serum magnesium in AA individuals using both genome-wide and candidate region interrogation approaches and 2) follow up on the GWAS findings from EA populations, to identify environmental factors that may modify the association between genetic loci and serum magnesium in both EA and AA populations.

We conducted a GWAS of serum magnesium in 2737 AA participants of the Atherosclerosis Risk in Communities (ARIC) Study and interrogated the regions of the nine published candidate loci from EA populations in the AA GWAS results. Index variants at loci that reached suggestive significance in AA participants of the ARIC study were put forward for replication in the Healthy Aging in Neighborhoods of Diversity across the Life Span (HANDLS) study. Gene-environment interaction analyses were conducted in the ARIC study for *MUC1* and *TRPM6* based on the results of literature search, which identified the influence of progesterone on *MUC1* expression and insulin on *TRPM6* expression.

## Results

### Study population characteristics

In the ARIC study, the sample sizes were 2,737 for AA participants and 8,926 for EA participants with mean serum magnesium levels of 0.79 mmol/L and 0.83 mmol/L respectively (Table 1). The HANDLS study included 942 AA participants with mean serum magnesium levels of 0.77 mmol/L.

**Table 1:** Study Participant Characteristics in the ARIC and HANDLS Studies

	ARIC study		HANDLS study
	European-Americans	African-Americans	African-Americans
N	8926	2737	942
Age, mean (SD)	54.3 (5.7)	53.4 (5.8)	48.3 (9.0)
Female, % (n)	53.0 (4728)	62.6 (1714)	55.9 (527)
Prevalent diabetes, % (n)	8.4 (749)	18.7 (513)	32.2 (303)
Diuretic use, % (n)	14.5 (1297)	28.8 (788)	13.6 (128)
Estimated glomerula filtration rate (mL/min/1.73 m^2^), mean (SD)	93.3 (12.7)	103.8 (18.0)	96.3 (24.1)
Serum Mg (mmol/L), mean (SD)	0.83 (0.07)	0.79 (0.08)	0.77 (0.08)

Abbreviation: SD, standard deviation

### Serum magnesium loci in ARIC African-American participants

**Fig. 1:**
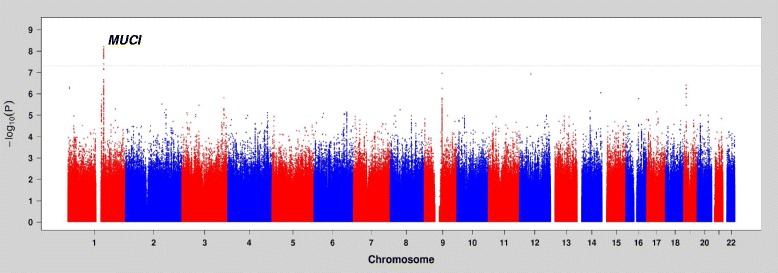
A plot of –log_10_(*P*-values) by genomic position of the GWAS results of serum magnesium in ARIC African-American participants

The GWAS in AA participants identified 17 SNPs near or at *MUC1* as genome-wide significant. The index SNP in ARIC AA participants was rs2974937 at *THBS3*, 2.7 kb upstream of *MUC1*, (beta = −0.013, frequency = 0.32, *p* = 6.1x10^−9^, Table 2). The index SNP previously identified in EA populations, rs4072037, was also genome-wide significant (beta = −0.014 mmol/L, frequency = 0.32, *p* = 2.2x10^−8^, Additional file 1: Table S1). The two index SNPs, rs4072037 and rs2974937, were in high linkage disequilibrium (r^2^ > 0.7 and D’ > 0.9 in ARIC EA and AA participants and the 1000 Genomes Phase 1 data, Additional file 1: Table S2). Fig. 1 presents the plot of –log_10_(*P*-values) by genomic position of the GWAS of serum magnesium in ARIC AA participants, and Additional file 1: Figure S1 and S2 present the quantile-quantile plot of the GWAS results and regional association plots of the *MUC1* locus, respectively.

**Table 2:** Association between Loci Identified in Populations of European Ancestry and Serum Magnesium in African-American Populations

					ARIC study African-American					HANDLS study African-American				Meta-analysis		
**Locus**	**Index SNP in ARIC AA participants**	**Chr**	**Position**	**Coded/Non-coded allele**	**Coded allele freq.**	**Beta**	**SE**	**P-Value**	**Imput. qual.**	**Coded allele freq.**	**Beta**	**P-Value**	**Imput. qual.**	**Beta**	**P-Value**	**P-Value threshold in region**
***MUC1***	rs2974937	1	155168849	C/T	0.32	−0.013	0.002	6.1E-09	0.95	0.35	−0.007	3.5E-02	0.99	−0.012	2.1E-11	8.9E-04
*MDS1*	rs74516251	3	168830777	A/G	0.11	−0.013	0.003	1.6E-04	0.99	0.11	−0.005	3.3E-01	0.97	−0.011	2.0E-05	4.9E-04
**SHROOM3**	rs9993810	4	77614640	A/G	0.47	−0.008	0.002	1.8E-04	0.92	0.48	−0.008	3.5E-02	0.93	−0.008	6.1E-06	7.4E-04
**TRPM6**	rs113607577	9	77455459	G/C	0.90	−0.019	0.004	1.1E-07	0.90	0.89	−0.012	4.7E-02	0.87	−0.017	4.6E-07	1.5E-03
*DCDC51*	rs7947033	11	30795987	C/T	0.10	−0.011	0.004	5.4E-03	0.74	0.08	−0.001	8.3E-01	0.91	−0.008	1.4E-02	1.0E-03
*ATP2B11*	rs36089488	12	90118300	T/C	0.95	−0.015	0.005	5.0E-03	0.78	0.94	−0.013	1.0E-01	0.85	−0.015	6.5E-04	9.8E-04
*HOXD91*	rs6745764	2	176974122	G/A	0.67	−0.008	0.002	9.5E-04	0.82	0.69	−0.005	1.9E-01	0.90	−0.007	3.1E-05	8.5E-04
*LUZP2*	rs11027876	11	24412973	G/C	0.18	−0.009	0.003	1.3E-03	0.86	0.18	−0.001	8.3E-01	0.89	−0.007	8.1E-03	6.4E-04
*PRMT7*	rs55857142	16	68236741	T/A	0.07	−0.018	0.004	2.7E-05	0.95	0.07	0.006	3.5E-01	0.97	−0.012	7.1E-04	9.4E-04

Betas are in mmol/L

Abbreviation: freq., frequency; Chr, chromosome; SNP, single nucleotide polymorphism; SE, standard error; imput. qual., imputation quality

Bold indicates significant association in African-American populations based on genome-wide or region-specific p-value threshold and replication in the HANDLS study. The I^2^, an indicator for heterogeneity between study, was 45 % for the index SNP at *MUC1*, 0 for *SHROOM3*, and 8 % for *TRPM6*

The index SNPs at eight other loci identified in EA populations did not have significant association (meta-analysis *p* > 0.01) with serum magnesium in AA participants of the ARIC and HANDLS studies (Additional file 1: Table S1). In the GWAS of ARIC AA participants, SNPs at five other loci showed suggestive significance (MAF ≥5 % and *p*-value <10^−6^, Additional file 1: Table S3). The index SNPs at these loci were not significantly associated with serum magnesium in the HANDLS study. The imputation quality of these index SNPs ranged from 0.58 to 0.97 in AA participants of the ARIC and HANDLS studies.

On the candidate region interrogation, the AA-specific index SNPs at five loci previously identified in EA populations (*MUC1*, *MDS1*, *SHROOM3*, *TRPM6*, and *PRMT7*) met the region-specific significant threshold (Table 2). The index SNPs at three of these five loci were replicated in the HANDLS study (*MUC1*, *SHROOM3*, and *TRPM6*). The AA-specific index SNPs at these three loci had imputation quality ≥ 0.87 in the AA participants of the ARIC and HANDLS studies. In addition, at *MUC1* and *TRPM6*, at least one genotyped SNP in ARIC AA participants was associated with serum magnesium below the region-specific threshold (Additional file 1: Table S4). Additional file 1: Figs. S2, S3, S4 present the regional association plots from ARIC AA participants for these three loci. Enhancement enrichment tests using HaploReg showed all three loci were enriched for enhancers in one or more cell lines (Additional file 1: Table S5).

### Effect modification between the index SNP at MUC1 and progestin use

The analysis for effect modification between *MUC1* and progestin use was conducted in EA participants only, because in the ARIC AA participants only 24 postmenopausal women reported the use of progestin. The most likely genotype of the index SNP at *MUC1* in EA populations, rs4072037, was independent of progestin use (Chi-square *p* = 0.21). In women with progestin use, the effect size of rs4072037 was more than two-fold larger (beta = −0.028 mmol/L, *p* = 7.3x10^−5^) than in other groups without progestin use (p for interaction = 0.01 to 0.05, Table 3). The phenotypic variance explained by rs4072037 in women with progestin use was 6.5 % versus ≤1.2 % for all other groups.

**Table 3:** Association Between rs4072037 C allele at *MUC1* and Serum Magnesium by Gender and Progestin Use Status in ARIC European Americans

	N	Beta (mmol/L)	SE	*P*-Value	Variance explained	*P*-Value for interaction
Male	4198	−0.009	0.002	7.5E-09	0.8 %	0.02
Female, premenopausal	1428	−0.012	0.003	2.8E-05	1.2 %	0.05
Female, postmenopausal with no hormone use	2495	−0.011	0.002	7.0E-08	1.1 %	0.03
Female, postmenopausal with non-progestin hormone use	574	−0.005	0.004	2.4E-01	0.2 %	0.01
Female, postmenopausal with progestin use	231	−0.028	0.007	7.3E-05	6.5 %	Reference

### Effect modification between the index SNPs at TRPM6 and fasting insulin

At *TRPM6*, the index SNP identified in EA populations [6], rs11144134, had very low frequency in ARIC AA participants (MAF = 2 %), and the index SNP in AA participants, rs113607577 (Table 2), was monomorphic in ARIC EA participants. Thus, the analysis for the effect modification between *TRPM6* and fasting insulin levels was conducted in EA and AA participants using the index SNP within the respective populations. The most likely genotype of the index SNPs in both cohorts was independent of insulin levels (Chi-square *p* = 0.77 in EA participants and 0.49 in AA participants).

In ARIC EA participants, the association between rs11144134 and serum magnesium levels was stronger in individuals with lower fasting insulin levels (<80 mmol/L, beta = −0.013, *p* = 1.6x10^−7^) than in those with higher fasting insulin levels (≥80 mmol/L, beta = −0.008, *p* = 0.02, p for interaction = 0.03, Table 4). Similarly, in ARIC AA participants, the association between rs113607577 and serum magnesium levels was also stronger in individuals with lower fasting insulin levels (<80 mmol/L, beta = −0.026, *p* = 3.5x10^−6^) than in those with higher fasting insulin levels (≥80 mmol/L, beta = −0.014, *p* = 9.0x10^−3^, p for interaction = 0.24).

**Table 4:** Association between Index SNPs at *TRPM6* and Serum Magnesium by Fasting Insulin Levels in ARIC European and African Americans

	N	Beta (mmol/L)	SE	*P*-Value	Variance explained	P for interaction
**European-Americans (rs11144134 T allele, frequency 0.92)**						
Fasting insulin < 80pmol/L	5937	−0.013	0.002	1.6E-07	0.44 %	0.03
Fasting insulin ≥ 80pmol/L	2752	−0.008	0.003	1.8E-02	0.19 %	
**African-Americans (rs113607577 G allele, frequency 0.90)**						
Fasting insulin < 80pmol/L	1186	−0.026	0.006	3.5E-06	1.55 %	0.24
Fasting insulin ≥ 80pmol/L	1302	−0.014	0.005	9.0E-03	0.58 %	

Two nonsynonymous SNPs at *TRPM6* were reported to impair the response of *TRPM6* to higher insulin levels (rs2274924 [K1584E] and rs3750425 [V1393I]) [9]. These two SNPs were in moderate linkage disequilibrium with the GWAS index SNPs at this locus (D’ between 0.44 to 0.74 in ARIC EA and AA participants, Additional file 1: Table S6). In ARIC EA participants, the association between these two nonsynonymous SNP with serum magnesium appeared to be independent of rs11144134, the GWAS index SNP in EA populations. In ARIC AA participants, these two nonsynonymous SNPs were not associated with serum magnesium levels (Additional file 1: Table S7).

## Discussion

### Main findings

Three of nine loci associated with serum magnesium previously discovered in EA populations were found to be associated in AA populations (*MUC1, TRPM6*, and *SHROOM3). MUC1* was genome-wide significant in ARIC AA participants. Together the index SNPs at these three loci explained 2.8 % of serum magnesium variation in ARIC AA participants. In addition, we showed that in ARIC EA participants, the associations of serum magnesium with *MUC1* and *TRPM6* were modified by progestin use and insulin levels respectively, extending the findings of functional experiments on gene expression to genetic association studies at the population level.

### In the context of the literature

The effect modification between the index SNP at *MUC1* and progestin use on serum magnesium levels is consistent with the known gene function of *MUC1* and supports *MUC1* as the causal gene at this locus. *MUC1* encodes a transmembrane mucin that forms part of the mucosal barrier in the intestine [10] and is also expressed in the kidney [11]. Higher progesterone levels increase the expression of *MUC1* in endometrial cells through increased promoter activity [12, 13]. The index SNPs in ARIC EA and AA participants were 6.7 kb apart and in high linkage disequilibrium, suggesting the two populations may share the same causal variant(s). The index SNP reported in EA populations (rs4072037), at <1 kb upstream of the transcription start site, is in a regulatory region [14] and in moderate linkage disequilibrium with an expression tag SNP (rs4971093) of *MUC1* [15]. The minor allele of the index SNP was associated with decreased serum magnesium levels with significantly stronger association in women with progestin use. A potential explanation could be that the role of *MUC1* as part of the intestinal mucosal barrier affects the paracellular absorption of magnesium, and that genetic effects on *MUC1* become more apparent when there is increased *MUC1* expression.

In contrast, *TRPM6* is a magnesium transporter involved in active magnesium absorption in the intestine and reabsorption in the distal convoluted tubule of the kidney [16, 17]. Higher insulin levels increase the expression of *TRPM6* mediated by insulin receptors [9]. Although the index SNPs at this locus in EA and AA participants were not in high linkage disequilibrium and more than 25 kb apart, the major alleles of both index SNPs were associated with lower serum magnesium levels. This association was attenuated in individuals with higher insulin levels. If higher insulin increases the transcription of *TRPM6* and leads to increase magnesium absorption, a potential explanation could be that the effect of the causal variant(s) at *TRPM6* is less apparent when there are more *TRPM6* receptors to transport magnesium. Since magnesium transport in kidney and intestine also occurs through additional routes and there are multiple ways by which higher insulin could influence serum magnesium levels, more complex explanations are also conceivable. The GWAS index SNPs in ARIC EA and AA participants were largely independent of the two nonsynonymous SNP found to influence *TRPM6* transcription levels [9]. This suggests that multiple functional variants associated with serum magnesium exist at *TRPM6*.

### Strengths and limitations

The strengths of this study include sufficient sample size for identifying the associations of serum magnesium loci previously discovered in EA populations in ARIC AA participants using a candidate region interrogation approach. We also have adequate sample size to assess gene-environment interaction in ARIC EA participants. Some limitations warrant mentioned. The suggestive significant serum magnesium loci identified in ARIC AA participants did not replicate in the HANDLS study, which could be due to a number of reasons, including limited power, moderate imputation quality, or type I error in the discovery sample. The association of the AA-specific index SNP at *SHROOM3* with serum magnesium has not been verified by genotyped SNPs, although the association was replicated in the HANDLS study. The interaction between *MUC1* and progestin use in ARIC EA participants was not assessed in another study. The interaction between *TRPM6* and insulin levels was significant in ARIC EA participants but not in ARIC AA participants. However, the direction of association was consistent between ARIC EA and AA participants. Post-hoc power analysis suggests the lack of significant association on the interaction between *TRPM6* and insulin levels in ARIC AA participants is likely due to low power.

## Conclusion

In summary, we identified three loci (*MUC1*, *SHROOM3*, and *TRPM6*) as associated with serum magnesium in African-Americans. In addition, we showed that two serum magnesium associated loci, *MUC1* and *TRPM6*, had significant effect modification with progestin use and insulin levels, respectively, in European Americans. These findings extend previous research on magnesium metabolism and demonstrate gene-environment interaction can be identified systematically by following up on findings from gene expression functional experiments.

## Methods

### Study population

The ARIC Study is a community-based prospective observational study of individuals between the ages of 45 and 64 years. Participants were drawn from a probability-based sample from 4 US communities (Forsyth County, NC; Jackson, MS; suburban Minneapolis, MN; and Washington County, MD). The baseline visit occurred between 1987 and 1989. Details of the ARIC cohort have been published elsewhere [18].

Of the total 15,792 participants in the ARIC study, 9,044 EA and 2,874 AA participants had genotypes that met quality control criteria for GWAS (Additional file 1: Supplementary Methods section). Participants with estimated glomerular filtration rate (eGFR) <30 mL/min/1.73 m^2^ (7 in EA, 97 in AA), missing diabetes status (12 in EA, 10 in AA), missing serum magnesium or having serum magnesium outside of 3 standard deviations within the specific population (99 in EA, 30 in AA) were not included in the analysis. As a result, the analyzed sample in the ARIC study included 8,926 EA participants and 2,737 AA participants. The threshold of 30 mL/min/1.73 m^2^ for eGFR was applied because the kidney’s ability to maintain serum magnesium levels within normal range deteriorates substantially when eGFR falls below this level [19]. Among EA participants, mean serum magnesium levels were slightly higher in those included in the analysis (0.83 mmol/L) than in those excluded (0.82 mmol/L, *p* = 0.003). Among AA participants, mean serum magnesium levels were similar between those included and excluded in the analysis (0.79 mmol/L in both).

The HANDLS study, the replication cohort for serum magnesium association in African-Americans, is a community-based, longitudinal study of socioeconomically diverse AA and EA individuals in Baltimore [20]. A total of 942 AA participants with imputed genotype and data in serum magnesium and covariates were included in the replication analysis.

### Serum magnesium and other measures

In the ARIC study, serum magnesium was measured using the metallochromic dye, Calmagite, based on the procedure of Gindler and Heth [21]. Prevalent diabetes mellitus was defined as a having a fasting glucose level at least 126 mg/dL, nonfasting glucose level at least 200 mg/dL, self-reported use of diabetes medication or self-reported physician diagnosis of diabetes. eGFR was calculated from standardized serum creatinine using the equation developed by the Chronic Kidney Disease Epidemiology Collaboration (CKD-EPI) [22]. Postmenopausal hormone use was determined based on inspection of medication bottles. Menopausal status was based on self-reported. Fasting serum insulin was measured using a radioimmunoassay (125Insulin Kit, Cambridge Medical Diagnostics, Billerica, Massachusetts) which was not specific for insulin and contained some cross-reactivity with pro-insulin. The details of the validity and reproducibility of this assay have been previously published [23].

In the HANDLS study, serum magnesium was measured using a spectrophotometry assay by Quest Diagnostics Nichols institute (Chantilly Virginia). Prevalent diabetes mellitus was defined as a having a fasting glucose level at least 126 mg/dL or self-reported of diabetes. eGFR was calculated from standardized serum creatinine using the CKD-EPI equation [22].

### Genotyping and imputation

Details of genotyping, quality control, and imputation are reported in the Additional file 1: Supplementary Methods section. Briefly, genotyping was performed using the Affymetrix Genome-wide Human SNP Array 6.0 in the ARIC study and the Illumina 1 M array in the HANDLS study. In both studies, individuals who were ancestry outlier or had cryptic relatedness were excluded. Imputation in AA participants in both studies was conducted using genotyped SNPs with MAF > 0.01, call rate > 0.95, and Hardy-Weinberg equilibrium *p*-value > 0.00001 and the 1000 Genomes March 2012 reference panels. The imputed genotype dosages were further filtered by MAF < 0.01 and imputation quality <0.3.

### Statistical methods for genome-wide association study and candidate Region interrogation

We conducted a GWAS in the ARIC AA participants using linear regression with serum magnesium as the outcome controlling for age, center, gender, prevalent diabetes status, eGFR and its squared term, diuretic use, and genotype-based principal components with significant association (*p* < 0.05) with serum magnesium. The genetic model was assumed to be additive. Genome-wide significance was defined by a p-value <5x10^−8^, and suggestive significance was defined by a *p*-value <10^−6^. For the nine index SNPs for serum magnesium previously identified in EA populations, the significance threshold was set at 0.05 divided by the number of SNPs (=0.006). Since allelic heterogeneity and differences in linkage disequilibrium pattern between EA and AA populations may give rise to different tag SNPs in an associated region, we evaluated the association of each locus previously identified in EA populations [6] in the GWAS results of ARIC AA participants based on a set of criteria similar to those developed in Liu *et al.* [24]. The region-specific significant threshold was defined as p-value <0.05 divided by the number of independent SNPs in the region of the locus. The span of the region was defined as between the two closest recombination hot spots on both sides of the published index SNP, or the length of gene containing the index SNP, whichever was larger. The number of independent SNPs at each locus was determined using PLINK [25] by pruning SNPs with variance inflation factor > 2 using a 50-SNP sliding window with a shift of 5 SNPs.

### Replication

The index SNPs with suggestive significance in the GWAS of ARIC AA participants and the ARIC AA-specific index SNPs in known EA loci were sent forth for replication in the HANDLS study using the same regression model. The significant criteria for replication were set as *p*-value < 0.05 with consistent direction of association. We performed fixed effect meta-analysis [26] combining the results from AA participants of the ARIC and HANDLS studies. A known EA locus was considered to be associated in AA populations if the AA-specific index SNP from the ARIC study met the replication criteria and the region-specific significance threshold.

### Enhancer enrichment test

Since loci associated with complex traits identified in GWAS tend to reside in regulatory regions [27], for the three AA-associated loci (*MUC1*, *SHROOM3*, and *TRPM6*) that were replicated in the HANDLS study, we conducted enhancer enrichment test using HaploReg version 2 [28]. At each locus, the SNPs used in the tests were those with D’ > 0.9 and r^2^ > 0.2 with the AA-specific index SNP based on 1000 Genomes Phase 1 AFR data and retrieved from HaploReg. The numbers of SNPs used were 58 at *MUC1*, 11 at *SHROOM3*, and 27 at *TRPM6*.

### Gene-environment interaction analyses for MUC1 and TRPM6

To investigate gene-environment interaction, we focused on *MUC1* and *TRPM6*. In addition to being the loci that had the largest effect estimates in EA participants, both loci are known to be involved in nutrient transport [10] with *TRPM6* being a magnesium transporter [16]. Their gene functions support them as the causal genes in the GWAS-identified loci. The index SNP at *MUC1* in EA populations, rs4072037, seems to be located in a regulatory region based on data from RegulomeDB [14] and in moderate linkage disequilibrium with an expression tag SNP of *MUC1* (rs4971093 at 155,144,300 bp) based on data from the Genotype-Tissue Expression (GTEx) project [15]. We searched the literature for factors that influence the expression levels of *MUC1* and *TRPM6* and found that progesterone regulates *MUC1* [12]*,* and insulin regulates *TRPM6* [9]*.* Hence we tested for the effect modification between progestin use and *MUC1* and between fasting insulin levels and *TRPM6* in relation to serum magnesium levels.

The analyses for gene-environment interaction were conducted within each self-reported race group in the following steps. First, the potential effect modifier was coded as a categorical variable. For progestin use, gender, postmenopausal status, and hormone use were combined into a categorical variable with 5 categories: males, premenopausal females, postmenopausal females with hormone therapy, postmenopausal females with non-progestin hormone therapy, and postmenopausal females with progestin use. Fasting insulin levels were categorized into two levels at 80 mmol/L, which is considered as a threshold indicating insulin resistance [29, 30]. Second, we tested for the independence between the most likely genotype of the index SNP and the potential effect modifier using Chi-square tests. Third, we performed linear regression using serum magnesium levels as the outcome, included the same covariates used in the GWAS of ARIC AA participants and tested for statistical interaction between the potential effect modifier and the index SNPs at the locus. Fourth, we evaluated the association between the index SNP and serum magnesium within each category of the effect modifier. For the evaluation of effect modification between insulin and the *TRPM6* index SNP, participants who did not fast for 8 hours before blood drawn (182 in EA and 187 in AA) or were taking insulin (55 in EA and 61 in AA) were excluded, resulting in a sample size of 8689 in EA participants and 2488 in AA participants.

In addition, two nonsynonymous SNPs (nsSNPs) at *TRPM6* were reported to inhibit the expression of *TRPM6* in response to higher insulin levels (rs2274924 [K1584E] and rs3750425 [V1393I]) [9]. To determine whether the association between the GWAS index SNPs and serum magnesium were independent of these two nsSNPs, we estimated the linkage disequilibrium as measured by D’ between the GWAS index SNPs and these two nsSNPs at *TRPM6.* D’ was used as the measure of linkage disequilibrium here because it estimates whether two variants are on the same haplotype across allele frequencies. Next, we evaluated the association between these two nsSNPs and serum magnesium by fasting insulin levels with and without controlling for the GWAS index SNP at *TRPM6*.

To assess the required sample size for replicating the gene-environment interaction in other cohorts, we conducted a post-hoc power analysis by simulating the distributions of genotypes and effect sizes found in ARIC EA participants. Using an alpha of 0.05, the power for detecting an interaction between the index SNP at *MUC1* and postmenopausal females with progestin use versus male was 27 % for a sample size of 3000, and 68 % for a sample size of 9000. For the interaction between the index SNP at *TRPM6* and insulin levels, the power was 34 % for a sample size of 3000, and 75 % for a sample size of 9000.

### Software

Linkage disequilibrium in ARIC study as measured by r^2^ and D’ was estimated using PLINK 1.07 [25] based on most likely genotypes derived from imputed dosage. The percentage of phenotypic variance explained by a SNP was estimated by calculating the effect size of the SNP (2β^2^xMAFx[1-MAF]) [31] divided by the phenotypic variance. Regression analyses were performed using the imputed dosage of the genotype. GWAS was conducted using SNPTEST 2.4.1 [32]. Fixed effect meta-analyses were conducted using metal [26]. Other analyses were performed using R. The present study has been approved by the respective Institutional Review Boards of the ARIC study and the HANDLS study and was performed in accordance with the Declaration of Helsinki. All participants provided informed consent.

### Availability of supporting data

The data set supporting the results of this article is available in the dbGaP repository, phs000090.v2.p1

(http://www.ncbi.nlm.nih.gov/projects/gap/cgi-bin/study.cgi?study_id=phs000090.v2.p1).

## Additional files

Additional file 1: Figure S1. Quantile-quantile plot of the P-values of the GWAS of serum magnesium in African-American participants in the ARIC study. Genomic control factor: 1.008. **Figure S2a and b**. Regional association plot of the *MUC1* locus from the GWAS in ARIC African Americans. 2A shows the closest recombination hot spots on both sides of the index SNP, and 2B provides a close-up view of the region. **Figure S3** Regional association plot of the *SHROOM3* locus in ARIC African Americans. **Figure S4** Regional association plot of the *TRPM6* locus in ARIC African Americans. **Table S1** Association of Index SNPs in published Loci of Serum Magnesium Identified in Populations of European Ancestry1 in African Americans in the ARIC and HANDLS Studies. **Table S2** Linkage Disequilibrium between the Index SNPs in Populations of European and African Ancestries at the *MUC1* Locus (rs4072037 and rs2974937). **Table S3** Novel Loci with Suggestive Significance (P-Value < 1e-7) in the GWAS of Serum Magnesium in ARIC African Americans with Replication Results from the HANDLS Study. **Table S4** Association between Genotyped SNPs in the *MUC1* and *TRPM6* Region with Serum Magnesium in ARIC African-Americans. **Table S5** Results of Enhancer Enrichment Tests at Three Replicated Serum Magnesium Loci (*MUC1*, *SHROOM3*, and *TRPM6*) of African Americans using HaploReg. **Table S6** Linkage Disequilibrium (D’) of GWAS Index SNPs and Two Nonsynonymous SNPs (rs2274924 and rs3750425) Reported to Influence *TRPM6* Expression1 at the *TRPM6* Locus in ARIC European- and African-Americans. **Table S7** Association between Two Nonsynonymous SNPs (rs2274924 and rs3750425) Reported to Influence *TRPM6* Expression1 and Serum Magnesium by Fasting Insulin Strata in ARIC European- and African-Americans. **Supplementary Methods**: Genotyping and Imputation in the ARIC study.
