# Supplementary material for: Genetic loci for serum magnesium among African-Americans and gene-environment interaction at MUC1 and TRPM6 in European-Americans: the Atherosclerosis Risk in Communities (ARIC) study
Source: BMC Genet. 2015 May 29;16:56. doi: 10.1186/s12863-015-0219-7 (PMC4462077; doi:10.1186/s12863-015-0219-7)
Supplement: Additional file 1: Figure S1. — Quantile-quantile plot of the P-values of the GWAS of serum magnesium in African-American participants in the ARIC study. Genomic control factor: 1.008. Figure S2a and b. Regional association plot of the MUC1 locus from the GWAS in ARIC African Americans. 2A shows the closest recombination hot spots on both sides of the index SNP, and 2B provides a close-up view of the region. Figure S3 Regional association plot of the SHROOM3 locus in ARIC African Americans. Figure S4 Regional association plot of the TRPM6 locus in ARIC African Americans. Table S1 Association of Index SNPs in published Loci of Serum Magnesium Identified in Populations of European Ancestry1 in African Americans in the ARIC and HANDLS Studies. Table S2 Linkage Disequilibrium between the Index SNPs in Populations of European and African Ancestries at the MUC1 Locus (rs4072037 and rs2974937). Table S3 Novel Loci with Suggestive Significance (P-Value < 1e-7) in the GWAS of Serum Magnesium in ARIC African Americans with Replication Results from the HANDLS Study. Table S4 Association between Genotyped SNPs in the MUC1 and TRPM6 Region with Serum Magnesium in ARIC African-Americans. Table S5 Results of Enhancer Enrichment Tests at Three Replicated Serum Magnesium Loci (MUC1, SHROOM3, and TRPM6) of African Americans using HaploReg. Table S6 Linkage Disequilibrium (D’) of GWAS Index SNPs and Two Nonsynonymous SNPs (rs2274924 and rs3750425) Reported to Influence TRPM6 Expression1 at the TRPM6 Locus in ARIC European- and African-Americans. Table S7 Association between Two Nonsynonymous SNPs (rs2274924 and rs3750425) Reported to Influence TRPM6 Expression1 and Serum Magnesium by Fasting Insulin Strata in ARIC European- and African-Americans. Supplementary Methods: Genotyping and Imputation in the ARIC study. [file 12863_2015_219_MOESM1_ESM.pdf]

**Supplementary Material for**

# Genetic Loci for Serum Magnesium among African-Americans and Gene-Environment Interaction at *MUC1* and *TRPM6* in European-Americans: the Atherosclerosis Risk in Communities (ARIC) Study

Adrienne Tin*, Anna Köttgen, Aaron R. Folsom, Nisa M. Maruthur, Mike A. Nalls, Michele K. Evans, Alan B. Zonderman, Christopher A. Friedrich, Eric Boerwinkle, Josef Coresh, Wen Hong Linda Kao

*To whom correspondence should be addressed. E-mail: atin1@jhu.edu

This file includes Supplementary Figures 1 to 4, Supplementary Tables 1 to 7, and Supplementary Methods.

**Supplementary Figure 1. Quantile-quantile plot of the P-values of the GWAS of serum magnesium in African-American participants in the ARIC study. Genomic control factor: 1.008**

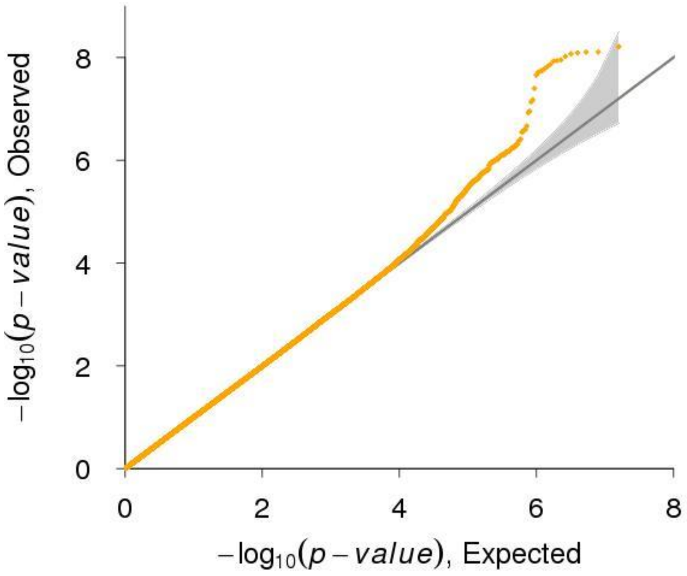

**Supplementary Figures 2A and B. Regional association plot of the *MUC1* locus from the GWAS in ARIC African Americans. 2A shows the closest recombination hot spots on both sides of the index SNP, and 2B provides a close-up view of the region.**

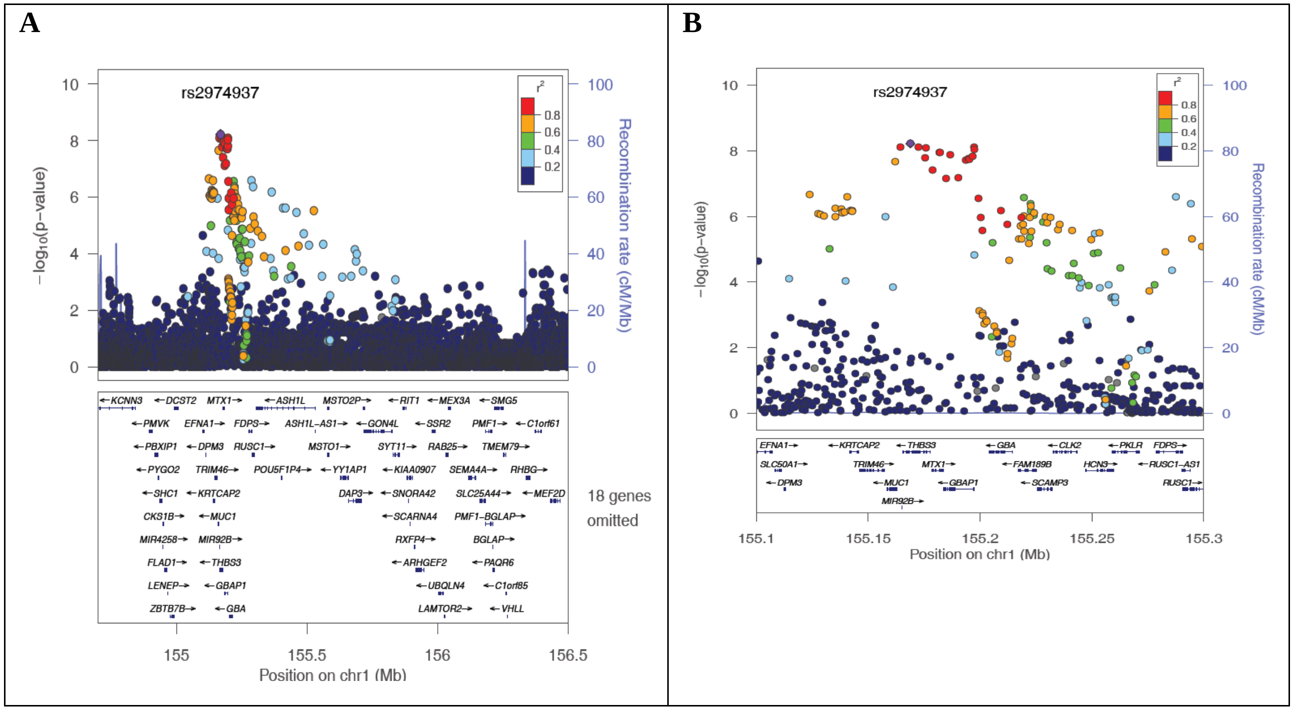

**Supplementary Figure 3. Regional association plot of the *SHROOM3* locus in ARIC African Americans**

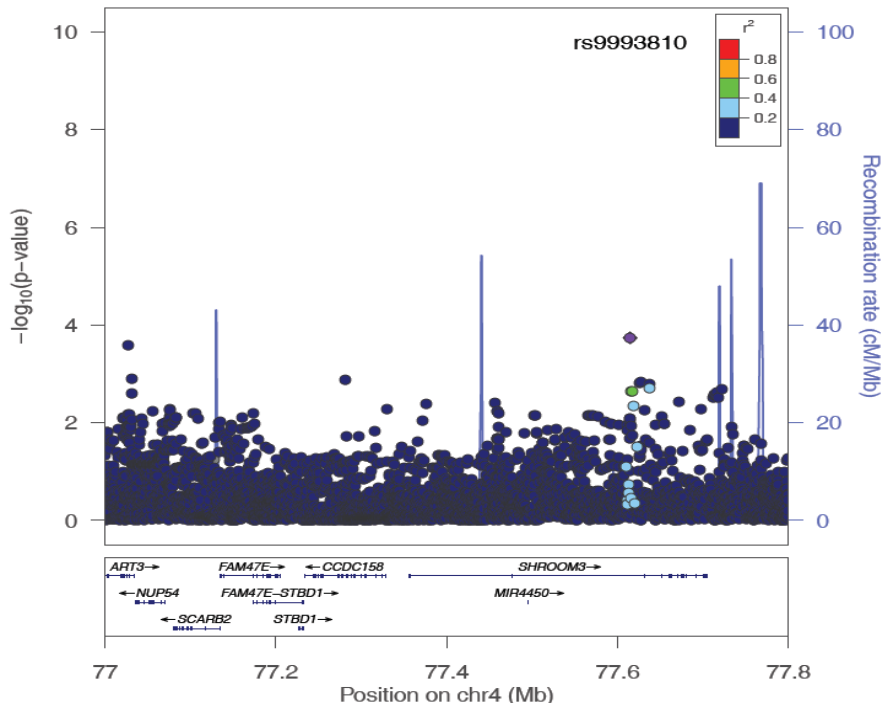

**Supplementary Figure 4. Regional association plot of the *TRPM6* locus in ARIC African Americans**

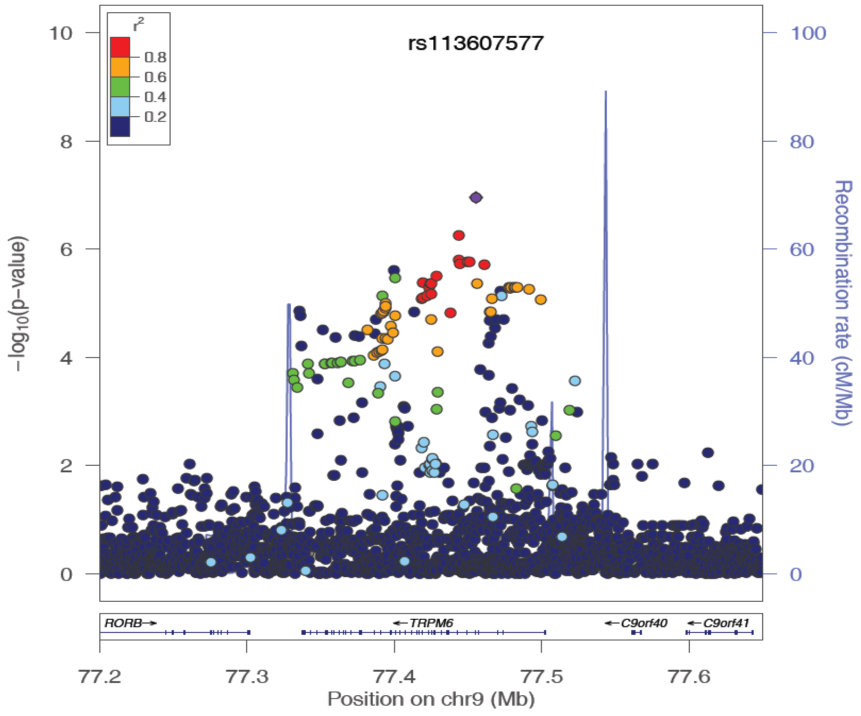

**Supplementary Table 1. Association of Index SNPs in published Loci of Serum Magnesium Identified in Populations of European Ancestry[1] in African Americans in the ARIC and HANDLS Studies**

| | | | | | ARIC Study African Americans | | | | | HANDLS Study African Americans | | | | Meta-analysis | |
|---|---|---|---|---|---|---|---|---|---|---|---|---|---|---|---|
| SNP | Chr | Position (b37) | Locus | Coded allelle | Coded allele Freq. | Beta | SE | P-Value | Imput. qual. | Coded Allele Freq. | Beta | P-Value | Imput. qual. | Beta | P-Value |
| rs4072037 | 1 | 155,162,067 | *MUC1* | C | 0.32 | -0.0136 | 0.002 | 2.2E-08 | 0.85 | 0.35 | -0.0065 | 6.7E-02 | 0.99 | -0.0119 | 8.8E-12 |
| rs2592394 | 2 | 176,991,779 | *HOXD91* | A | 0.20 | -0.0048 | 0.003 | 8.7E-02 | 0.86 | 0.19 | -0.0003 | 9.5E-01 | 0.99 | 0.0034 | 1.7E-01 |
| rs448378 | 3 | 169,100,899 | *MDS1* | G | 0.51 | -0.0024 | 0.002 | 2.6E-01 | 1.00 | 0.51 | -0.0022 | 5.2E-01 | 1.00 | -0.0024 | 1.8E-01 |
| rs13146355 | 4 | 77,412,140 | *SHROOM3* | G | 0.86 | -0.0032 | 0.003 | 3.1E-01 | 0.97 | 0.86 | 0.0014 | 7.9E-01 | 0.98 | 0.0021 | 4.3E-01 |
| rs11144134 | 9 | 77,499,796 | *TRPM6* | T | 0.98 | -0.0042 | 0.008 | 6.1E-01 | 0.97 | 0.98 | -0.0095 | 4.4E-01 | 0.92 | 0.0058 | 3.9E-01 |
| rs4561213 | 11 | 24,678,819 | *LUZP2* | G | 0.63 | 0.0000 | 0.002 | 9.9E-01 | 0.98 | 0.64 | 0.0032 | 3.7E-01 | 0.98 | 0.0008 | 6.7E-01 |
| rs3925584 | 11 | 30,760,335 | *DCDC51* | C | 0.12 | -0.0070 | 0.003 | 2.9E-02 | 0.98 | 0.11 | -0.0048 | 3.8E-01 | 1.00 | -0.0065 | 1.4E-02 |
| rs7965584* | 12 | 90,305,779 | *ATP2B11* | T | 0.09 | -0.0114 | 0.004 | 1.2E-01 | 1.00 | -- | -- | -- | -- | -- | -- |
| rs7197653 | 16 | 68,383,047 | *PRMT7* | C | 0.35 | 0.0003 | 0.002 | 8.9E-01 | 0.94 | 0.31 | -0.0012 | 7.6E-01 | 0.93 | 0.0005 | 7.8E-01 |

*The SNP rs7965584 was not present in the 1000G reference panels. In HapMap release 22, rs10858938 (4100 bp away) was the closest SNP with an R-square of 0.96 and D' of 1 with rs7965584 and was therefore used as the proxy to assess the association between rs7965584 and serum magnesium in ARIC African American participants.

The $I^2$, an indicator of heterogeneity between studies was 68% for the index SNP at *MUC1* and 0 for all other SNPs.

Abbreviation: Freq., frequency; imput. qual., imputation quality; Chr, chromosome; SNP, single nucleotide polymorphism; SE, standard error

Regression covariates included age, sex, center, prevalent diabetes, eGFR and its square term, and the use of diuretics.

[1]Meyer, TE, Verwoert, GC, Hwang, SJ, Glazer, NL, Smith, AV, van Rooij, FJ, Ehret, GB, Boerwinkle, E, Felix, JF, Leak, TS, Harris, TB, Yang, Q, Dehghan, A, Aspelund, T, Katz, R, Homuth, G, Kocher, T, Rettig, R, Ried, JS, Gieger, C, Prucha, H, Pfeufer, A, Meitinger, T, Coresh, J, Hofman, A, Sarnak, MJ, Chen, YD, Uitterlinden, AG, Chakravarti, A, Psaty, BM, van Duijn, CM, Kao, WH, Witteman, JC, Gudnason, V, Siscovick, DS, Fox, CS, Kottgen, A: Genome-wide association studies of serum magnesium, potassium, and sodium concentrations identify six Loci influencing serum magnesium levels. *PLoS Genet*, 6, 2010.

**Supplementary Table 2. Linkage Disequilibrium between the Index SNPs in Populations of European and African Ancestries at the *MUC1* Locus (rs4072037 and rs2974937)**

| | ARIC | | 1000 Genomes Phase 1 | |
|---|---|---|---|---|
| | European-Americans | African-Americans | EUR | AFR |
| **D'** | 1.00 | 0.95 | 1.00 | 0.91 |
| **$r^2$** | 0.94 | 0.85 | 0.94 | 0.72 |

rs4072037 was the index SNP identified in populations of European ancestry in Meyer et al. 2010.
rs2974937 was the index SNP identified in ARIC African-Americans.
The linkage disequilibrium information from the ARIC study was calculated based on most likely genotype using PLINK.
The linkage disequilibrium information from the 1000 Genomes Phase 1 project was retrieved from HaploReg version 2.

**Supplementary Table 3. Novel Loci with Suggestive Significance (P-Value < 1e-7) in the GWAS of Serum Magnesium in ARIC African Americans with Replication Results from the HANDLS Study**

| | | | | | ARIC African Americans | | | | | HANDLS Study | | | | Meta-analysis | |
|---|---|---|---|---|---|---|---|---|---|---|---|---|---|---|---|
| SNP | Chr | Position | Locus | Coded /Non-coded Allele | Coded allele freq. | Beta | SE | pvalue | Imput. qual. | Coded allele freq. | Beta | P-Value | Imput. qual. | Beta | P-Value |
| rs112798180 | 1 | 7507389 | CAMTA1 | C/G | 0.06 | -0.025 | 0.005 | 4.9E-07 | 0.81 | 0.04 | 0.0039 | 0.73 | 0.58 | -0.020 | 1.1E-05 |
| rs74590795 | 4 | 86035397 | WDFY3 | T/A | 0.05 | -0.049 | 0.010 | 7.9E-07 | 0.97 | 0.05 | -0.0011 | 0.90 | 0.95 | -0.022 | 9.9E-04 |
| rs193153567 | 12 | 48946231 | OR8S1 | T/C | 0.10 | -0.021 | 0.004 | 1.2E-07 | 0.77 | 0.10 | -0.0069 | 0.31 | 0.66 | -0.017 | 4.7E-07 |
| rs915364 | 14 | 101598942 | MIR379 | A/C | 0.57 | 0.011 | 0.002 | 8.9E-07 | 0.93 | 0.55 | 0.0030 | 0.40 | 0.94 | 0.009 | 1.9E-07 |
| chr19:11751304:D | 19 | 11751304 | ZNF627 | C/CAT | 0.14 | 0.017 | 0.003 | 3.9E-07 | 0.81 | 0.14 | 0.0001 | 0.99 | 0.81 | 0.013 | 7.1E-07 |

The $I^2$, an indicator of heterogeneity between studies ranged from 69% to 92%.

Betas are in mmol/L.

Abbreviation: Freq., frequency; imput. qual., imputation quality; Chr, chromosome; SNP, single nucleotide polymorphism; SE, standard error

Regression covariates included age, sex, center, prevalent diabetes, eGFR and its square term, and the use of diuretics.

**Supplementary Table 4. Association between Genotyped SNPs in the *MUC1* and *TRPM6* Region with Serum Magnesium in ARIC African-Americans**

| Locus | Index SNP in ARIC AA | Chr | Position | P-Value | Genotyped SNP with lowest p-value | Position | Coded/ Noncoded allele | Coded allele freq. | Beta | SE | P-Value | P-Value threshold in region | $r^2$ | D' |
|---|---|---|---|---|---|---|---|---|---|---|---|---|---|---|
| *MUC1* | rs2974937 | 1 | 155168849 | 6.1E-09 | rs2990245 | 155197462 | C/T | 0.32 | -0.013 | 0.002 | 9.5E-09 | 8.9E-04 | 0.92 | 0.97 |
| *TRPM6* | rs113607577 | 9 | 77455459 | 1.1E-07 | rs11144094 | 77429569 | C/T | 0.81 | -0.009 | 0.003 | 4.3E-04 | 1.5E-03 | 0.47 | 0.92 |

The linkage disequilibrium information (r2 and D') between the index SNP and the genotyped SNP were based on 1000 Genomes Phase 1 AFR data retrieved from HaploReg version 2.
Each region is defined as between two recombination hot spots or 500kb on both sides of the index SNP whichever is smaller.

**Supplementary Table 5. Results of Enhancer Enrichment Tests at Three Replicated Serum Magnesium Loci (*MUC1*, *SHROOM3*, and *TRPM6*) of African Americans using HaploReg**

| Cell line ID | Cell line | P-Value for enrichment of the strongest enhancers |
|---|---|---|
| ***MUC1*** | | |
| H1 | H1 Cell Line | 4.0E-06 |
| HepG2 | Hepatocellular carcinoma | 5.4E-04 |
| HSMM | Skeletal muscle myoblasts | 3.2E-02 |
| HMEC | Mammary epithelial cells | 6.2E-05 |
| ***SHROOM3*** | | |
| H1 | H1 Cell Line | 8.0E-06 |
| Huvec | Umbilical vein endothelial cells | 1.8E-03 |
| HSMM | Skeletal muscle myoblasts | 1.8E-03 |
| HMEC | Mammary epithelial cells | 2.7E-02 |
| ***TRPM6*** | | |
| HMEC | Mammary epithelial cells | 3.5E-03 |

The SNPs used in the enhancer enrichment tests at each locus were those with D' > 0.9 and $r^2$ > 0.2 with the index SNP based on 1000 Genomes Phase 1 AFR data and retrieved from HaploReg. The numbers of SNPs used were 58 at *MUC1*, 11 at *SHROOM3*, and 27 at *TRPM6*.

**Supplementary Table 6. Linkage Disequilibrium (D') of GWAS Index SNPs and Two Nonsynonymous SNPs (rs2274924 and rs3750425) Reported to Influence *TRPM6* Expression[1] at the *TRPM6* Locus in ARIC European- and African-Americans**

| | | | | D' between SNPs | | |
|---|---|---|---|---|---|---|
| **SNP** | **Position** | **MAF in ARIC** | **Imputation Quality** | **rs2274924 (K1584E)** | **rs3750425 (V1393I)** | **rs11144134** |
| **European Americans** | | | | | | |
| **rs2274924 (K1584E)** | 77376647 | 0.16 | 1.00 | 1.00 | 1.00 | 0.57 |
| **rs3750425 (V1393I)** | 77377410 | 0.09 | 1.00 | | 1.00 | 0.44 |
| **rs11144134*** | 77499796 | 0.08 | 0.97 | | | 1.00 |
| | | | | **rs2274924 (K1584E)** | **rs3750425 (V1393I)** | **rs113607577** |
| **African Americans** | | | | | | |
| **rs2274924 (K1584E)** | 77376647 | 0.41 | 1.00 | 1.00 | 0.87 | 0.74 |
| **rs3750425 (V1393I)** | 77377410 | 0.38 | 1.00 | | 1.00 | 0.56 |
| **rs113607577**** | 77455459 | 0.10 | 0.90 | | | 1.00 |

*GWAS index SNP in populations of European ancestry
** GWAS index SNP in ARIC African-Americans

D’ indicates whether two variants are likely to reside on the same haplotype across allele frequencies.
D’ measures were estimated using the most likely genotype derived from the imputed genotype dosage.

Abbreviation: SNP, single nucleotide polymorphism; MAF, minor allele frequency

[1]Nair, AV, Hocher, B, Verkaart, S, van Zeeland, F, Pfab, T, Slowinski, T, Chen, YP, Schlingmann, KP, Schaller, A, Gallati, S, Bindels, RJ, Konrad, M, Hoenderop, JG: Loss of insulin-induced activation of TRPM6 magnesium channels results in impaired glucose tolerance during pregnancy. Proceedings of the National Academy of Sciences of the United States of America, 109: 11324-11329, 2012.

**Supplementary Table 7. Association between Two Nonsynonymous SNPs (rs2274924 and rs3750425) Reported to Influence *TRPM6* Expression[1] and Serum Magnesium by Fasting Insulin Strata in ARIC European- and African-Americans**

| SNP | Coded / noncoded Allele | Coded allele frequency | Beta | SE | P-Value | Coded allele frequency | Beta | SE | P-Value | P-Value for interaction |
|---|---|---|---|---|---|---|---|---|---|---|
| **European Americans** | | **Fasting insulin <80pmol/L (n=5937)** | | | | **Fasting insulin <80pmol/L (n=2752)** | | | | |
| rs2274924 (K1584E) | T/C | 0.84 | 0.005 | 0.002 | 2.1E-03 | 0.83 | -0.001 | 0.002 | 8.2E-01 | 0.05 |
| rs3750425 (V1393I) | C/T | 0.91 | 0.006 | 0.002 | 2.9E-03 | 0.91 | -0.001 | 0.003 | 8.0E-01 | 0.06 |
| ***rs11144134 and rs2274924 adjusted for each other*** | | | | | | | | | | |
| rs11144134* | T/C | 0.92 | -0.013 | 0.002 | 1.8E-08 | 0.93 | -0.005 | 0.004 | 2.0E-01 | 0.04 |
| rs2274924 (K1584E) | T/C | 0.84 | 0.005 | 0.002 | 7.2E-03 | 0.83 | -0.001 | 0.002 | 7.3E-01 | 0.067 |
| ***rs11144134 and rs3750425 adjusted for each other*** | | | | | | | | | | |
| rs11144134* | T/C | 0.92 | -0.013 | 0.002 | 1.2E-08 | 0.93 | -0.004 | 0.004 | 2.1E-01 | 0.035 |
| rs3750425 (V1393I) | C/T | 0.91 | 0.006 | 0.002 | 6.7E-03 | 0.91 | -0.001 | 0.003 | 7.6E-01 | 0.075 |
| **African Americans** | | **Fasting insulin <80pmol/L (n=1186)** | | | | **Fasting insulin <80pmol/L (n=1303)** | | | | |
| rs2274924 (K1584E) | T/C | 0.59 | -0.002 | 0.003 | 5.7E-01 | 0.59 | 0.001 | 0.003 | 7.7E-01 | 0.51 |
| rs3750425 (V1393I) | C/T | 0.72 | -0.0003 | 0.003 | 9.4E-01 | 0.72 | 0.002 | 0.003 | 4.6E-01 | 0.53 |

*GWAS index SNP in populations of European ancestry

Betas are in mmol/L.
Regression covariates include age, sex, center, prevalent diabetes, eGFR and its square term, and use of diuretics.

Abbreviation: SNP, single nucleotide polymorphism; SE, standard error

[1]Nair, AV, Hocher, B, Verkaart, S, van Zeeland, F, Pfab, T, Slowinski, T, Chen, YP, Schlingmann, KP, Schaller, A, Gallati, S, Bindels, RJ, Konrad, M, Hoenderop, JG: Loss of insulin-induced activation of TRPM6 magnesium channels results in impaired glucose tolerance during pregnancy. Proceedings of the National Academy of Sciences of the United States of America, 109: 11324-11329, 2012.

**Supplementary Methods**

**Genotyping and Imputation in the ARIC study**

The genotyping in the European American (EA) cohort was supported by the National Institute of Health Gene Environment Association Studies (GENEVA) project and used the Affymetrix Genome-wide Human SNP Array 6.0. The initial quality filters included a call rate of 0.95, check for blind duplicates in each plate and gender mismatch. Of the 910,030 single nucleotide polymorphisms (SNPs) genotyped, 839,048 passed the initial quality filters. Of the SNPs that failed the initial quality filters, 96% were due to call rates. After the genotype data was received from GENEVA (N=9,713), additional quality filters were applied to exclude individuals who were more than 8 standard deviations away from any of the 10 principal components or closely related based on identity by state distance (DST >0.86) calculated by PLINK.[1] Altogether 658 individuals were excluded resulting in a genotype dataset of 9044 individuals.

Genotype imputation was performed using IMPUTE2[2] after using ShapeIt (v1.r532)[3] for haplotype phasing. The imputation reference panels were from 1000 Genomes Phase I Integrated Release Version 3. The criteria for SNPs to be included in imputation are Hardy-Weinberg equilibrium (HWE) p-value >1e-5, SNP missingness <0.05, minor allele frequency (MAF) >0.005. This resulted in 711,589 SNPs used in imputation.

The genotyping platform and quality filters for African Americans (AA) were the same as those for the EA cohort, except for the criteria for relatedness exclusion and SNPs included in imputation. For relatedness, individuals were excluded based on identity by state distance (DST >0.8) calculated by PLINK[1] or 6 SD away from any of the first 10 principal components generated based on 391,965 autosomal SNPs with MAF >0.1, call rate >0.95 and HWE p-value >0.001. This resulted in at a genotype dataset of 2874 individuals. The criteria for SNPs to be included in imputation were HWE p-value >1e-5, SNP missingness <0.05, MAF >0.01, resulting in 806,416 SNPs included in imputation.

For the GWAS of ARIC AA participants, we included SNPs with MAF > 0.01 and imputation quality > 0.3, resulting in 14,825,944 SNPs. Principal components for capturing sub-population stratification were generated using EIGENSTRAT[4] in EA and AA participants separately. SNPs used for principal components generation were restricted to those with MAF ≥10%, missing data per SNP <0.5%, and HWE p-value ≥0.001.

**Genotyping and Imputation in the HANDLS study**

Genotypes were obtained using Illumina 1M array. Imputation was performed using miniMac[2] based on 1000 Genomes March 2012 reference panels. Individuals who were ancestry outliers and had cryptic relatedness were removed. The criteria for SNPs to be included in imputation were MAF>0.01,

call rate >0.95, and HWE p-value >0.00001, resulting in 907,763 SNPs. Imputed genotypes were further filtered by MAF <0.01 and imputation quality <0.3. The software packages for data management and statistical analysis were PLINK[1] and R.

## References


1. Purcell, S, Neale, B, Todd-Brown, K, Thomas, L, Ferreira, MA, Bender, D, Maller, J, Sklar, P, de Bakker, PI, Daly, MJ, Sham, PC: PLINK: a tool set for whole-genome association and population-based linkage analyses. *Am J Hum Genet*, 81**:** 559-575, 2007.
2. Howie, B, Fuchsberger, C, Stephens, M, Marchini, J, Abecasis, GR: Fast and accurate genotype imputation in genome-wide association studies through pre-phasing. *Nat Genet*, 44**:** 955-959, 2012.
3. Delaneau, O, Zagury, JF, Marchini, J: Improved whole-chromosome phasing for disease and population genetic studies. *Nat Methods*, 10**:** 5-6, 2013.
4. Price, AL, Patterson, NJ, Plenge, RM, Weinblatt, ME, Shadick, NA, Reich, D: Principal components analysis corrects for stratification in genome-wide association studies. *Nat Genet*, 38**:** 904-909, 2006.
